# Efficiency of Computer-Aided Facial Phenotyping (DeepGestalt) in Individuals With and Without a Genetic Syndrome: Diagnostic Accuracy Study

**DOI:** 10.2196/19263

**Published:** 2020-10-22

**Authors:** Jean Tori Pantel, Nurulhuda Hajjir, Magdalena Danyel, Jonas Elsner, Angela Teresa Abad-Perez, Peter Hansen, Stefan Mundlos, Malte Spielmann, Denise Horn, Claus-Eric Ott, Martin Atta Mensah

**Affiliations:** 1 Institute of Medical Genetics and Human Genetics Charité - Universitätsmedizin Berlin corporate member of Freie Universität Berlin, Humboldt-Universität zu Berlin and Berlin Institute of Health Berlin Germany; 2 Institute for Genomic Statistics and Bioinformatics University Hospital Bonn Rheinische Friedrich-Wilhelms-Universität Bonn Bonn Germany; 3 Klinik für Pädiatrie mit Schwerpunkt Gastroenterologie, Nephrologie und Stoffwechselmedizin Charité - Universitätsmedizin Berlin corporate member of Freie Universität Berlin, Humboldt-Universität zu Berlin and Berlin Institute of Health Berlin Germany; 4 Berlin Center for Rare Diseases Charité - Universitätsmedizin Berlin corporate member of Freie Universität Berlin, Humboldt-Universität zu Berlin and Berlin Institute of Health Berlin Germany; 5 The Jackson Laboratory for Genomic Medicine Farmington, CT United States; 6 RG Development & Disease Max Planck Institute for Molecular Genetics Berlin Germany; 7 Institute of Human Genetics University of Lübeck Lübeck Germany; 8 Berlin Institute of Health Berlin Germany

**Keywords:** facial phenotyping, DeepGestalt, facial recognition, Face2Gene, medical genetics, diagnostic accuracy, genetic syndrome, machine learning

## Abstract

**Background:**

Collectively, an estimated 5% of the population have a genetic disease. Many of them feature characteristics that can be detected by facial phenotyping. Face2Gene CLINIC is an online app for facial phenotyping of patients with genetic syndromes. DeepGestalt, the neural network driving Face2Gene, automatically prioritizes syndrome suggestions based on ordinary patient photographs, potentially improving the diagnostic process. Hitherto, studies on DeepGestalt’s quality highlighted its sensitivity in syndromic patients. However, determining the accuracy of a diagnostic methodology also requires testing of negative controls.

**Objective:**

The aim of this study was to evaluate DeepGestalt's accuracy with photos of individuals with and without a genetic syndrome. Moreover, we aimed to propose a machine learning–based framework for the automated differentiation of DeepGestalt’s output on such images.

**Methods:**

Frontal facial images of individuals with a diagnosis of a genetic syndrome (established clinically or molecularly) from a convenience sample were reanalyzed. Each photo was matched by age, sex, and ethnicity to a picture featuring an individual without a genetic syndrome. Absence of a facial gestalt suggestive of a genetic syndrome was determined by physicians working in medical genetics. Photos were selected from online reports or were taken by us for the purpose of this study. Facial phenotype was analyzed by DeepGestalt version 19.1.7, accessed via Face2Gene CLINIC. Furthermore, we designed linear support vector machines (SVMs) using Python 3.7 to automatically differentiate between the 2 classes of photographs based on DeepGestalt's result lists.

**Results:**

We included photos of 323 patients diagnosed with 17 different genetic syndromes and matched those with an equal number of facial images without a genetic syndrome, analyzing a total of 646 pictures. We confirm DeepGestalt’s high sensitivity (top 10 sensitivity: 295/323, 91%). DeepGestalt’s syndrome suggestions in individuals without a craniofacially dysmorphic syndrome followed a nonrandom distribution. A total of 17 syndromes appeared in the top 30 suggestions of more than 50% of nondysmorphic images. DeepGestalt’s top scores differed between the syndromic and control images (area under the receiver operating characteristic [AUROC] curve 0.72, 95% CI 0.68-0.76; *P*<.001). A linear SVM running on DeepGestalt’s result vectors showed stronger differences (AUROC 0.89, 95% CI 0.87-0.92; *P*<.001).

**Conclusions:**

DeepGestalt fairly separates images of individuals with and without a genetic syndrome. This separation can be significantly improved by SVMs running on top of DeepGestalt, thus supporting the diagnostic process of patients with a genetic syndrome. Our findings facilitate the critical interpretation of DeepGestalt’s results and may help enhance it and similar computer-aided facial phenotyping tools.

## Introduction

### Background

Although individual genetic diseases are rare, they collectively affect an estimated 5% of a population [1]. Thus, these diseases represent a major challenge for health care systems, as it usually requires highly specialized knowledge to propose a specific genetic diagnosis. Assessing the facial phenotypes of patients with genetic syndromes is key to this diagnostic process [2]. Traditionally performed by a physician, the advents of computer vision and machine learning in medicine enable rapid and automated assessment of a patient's facial traits [3,4]. Numerous facial phenotyping systems have been developed with the potential to aid the diagnostic processes in medical genetics [5-12]. DeepGestalt, the neural network behind Face2Gene CLINIC, which was trained on more than 17,106 images, is thus far the best-investigated and most convenient to use application [11]. Several studies assessed the algorithm's sensitivity, suggesting that it is of a certain quality [11,13-38]. These tests predominantly analyzed images of patients diagnosed with a genetic disorder known to show characteristic facial features. This appears reasonable as DeepGestalt is designed to identify such syndromes. However, it might introduce a bias in conclusions of the system's everyday clinical use since not all individuals seen in a real-life setting belong to the group of patients included in previous studies of DeepGestalt. This may be because (1) the featured syndrome is yet to be analyzed by the system; (2) an individual features a syndrome not associated with a characteristic facies; or (3) an individual has no syndrome at all.

In addition to such evaluations of DeepGestalt's sensitivity, there is a need for studies on its specificity when tested on individuals without craniofacial dysmorphism. As DeepGestalt is not designed to suggest the class label “inconspicuous face” [11], evaluating its clinical specificity is not too trivial a task. Some studies tested the ability of DeepGestalt's methodology to distinguish between facial images with and without a genetic syndrome by constructing user-specific neural networks trained on healthy control images and on images of limited numbers of well-selected genetic disorders using Face2Gene RESEARCH [20,26-28,30,32,34,39-41]. Their results suggested that neural networks such as DeepGestalt may have the potential to differentiate between the 2 classes and may thus be used in diagnosing patients in medical genetics. Such a test could be applied at different stages of the diagnostic process. Patients who want to know if genetic counseling is necessary could use it as a triage test to check whether a suspicion of a genetic disease is justified. Physicians and other medical professionals could similarly use such a test on patients suspected of having a genetic syndrome to narrow down the range of possible diagnoses. Geneticists could use it as an add-on test to further confirm a diagnosis, for example, in the presence of a variant of unknown significance.

### Objectives

We aimed to systematically benchmark DeepGestalt’s power to discern images of individuals with a dysmorphic genetic syndrome from images of healthy control individuals. For this purpose, we tested the basic prerequisite for the diagnostic usefulness of DeepGestalt, that is, to yield different scores in persons with a conventionally established diagnosis of a genetic syndrome than in persons without a genetic syndrome (H_1_: µ_syndromic_ ≠ µ_healthy_). We also determined DeepGestalt’s capacity to distinguish those images by measuring its area under the receiver operating characteristic (AUROC) curve. Furthermore, we aimed to develop and test a machine learning–based approach to improve DeepGestalt's accuracy.

## Methods

### Selection and Analysis of Portrait Photos

#### Study Design

To be included in this study, portrait photos had to depict the entire frontal face (from hairline to chin showing both eyes) and no artifact other than glasses. To achieve a vertical positioning of the face, the images were cropped and rotated if necessary. A convenience sample of online accessible images was collected between September 2019 and December 2019, using a methodology adjusted from Ferry et al [8]. Pictures photographed by us were taken at the 2018 meeting of the Elterninitiative Apertsyndrom und Verwandte Fehlbildungen eV, a parents’ initiative on Apert syndrome and related disorders in Germany, after obtaining written informed consents as approved by the ethics committee of the Charité – Universitätsmedizin Berlin (EA2/190/16). Image inclusion was planned before conducting analysis by DeepGestalt. A sample size of the positive and negative class of 105 (N=210) was calculated using G*Power, version 3.1.9.7 (effect size 0.5; α=.05; power 0.95; allocation ratio 1).

#### Defining Reference Phenotypes

Only images of individuals reported to be clinically or molecularly diagnosed with a genetic syndrome were labeled as syndromic. When no syndrome was reported and no facial gestalt suggestive of a syndrome was observed, as judged by physicians working in medical genetics, images were labeled as “healthy.”

#### Computer-Aided Facial Phenotyping

Computer-aided facial phenotyping was performed using DeepGestalt version 19.1.7, accessed via Face2Gene CLINIC (FDNA Inc). Neither the class labels nor diagnoses were passed to DeepGestalt. No other phenotypic information but 1 portrait photo per case was entered into the system. DeepGestalt's training set was tested not to contain duplicates of images used in this study, as described previously [42].

#### Danyel Cohort

The Danyel cohort, originally described by Danyel et al [30], comprises 116 healthy control images.

#### Syndromic Cohort

This cohort comprises frontal facial images of 17 syndromes. We planned to collect the same number of images for each of these syndromes. A total of 16 of these syndromes were chosen from the 201 distinct suggestions in DeepGestalt’s top 30 results lists of the Danyel cohort. Syndromes of different frequencies ranging from 76% (frequently suggested) to 1% (rarely suggested) were selected. In descending order of frequency, these syndromes are as follows: Fragile X syndrome (OMIM: #300624), Angelman syndrome (OMIM: #105830), Rett syndrome (OMIM: #312750), Phelan-McDermid syndrome (OMIM: #606232), Klinefelter syndrome, Beckwith-Wiedemann syndrome (OMIM: #130650), 22q11.2 deletion syndrome (OMIM: #611867), Sotos syndrome (OMIM: #117550), Noonan syndrome (OMIM: PS163950), Loeys-Dietz syndrome (OMIM: PS609192), Williams-Beuren syndrome (OMIM: #194050), Rubinstein-Taybi syndrome (OMIM: PS180849), achondroplasia (OMIM: #100800), Wolf-Hirschhorn syndrome (OMIM: #194190), Pallister-Killian syndrome (OMIM: #601803), and Treacher Collins syndrome (OMIM: PS154500). In addition, we chose Apert syndrome (OMIM: #101200), which was not implied in the Danyel cohort.

#### Matched Control Cohort

Each photo of the syndromic cohort was matched to an image of an individual without a genetic syndrome by age, sex, and ethnicity to build a cohort of an equal number of control images.

### Statistical Evaluation and Classification Experiments

Face2Gene CLINIC returns DeepGestalt’s top 30 syndrome suggestions. DeepGestalt associates each suggestion with a Gestalt score [11]. The syndrome suggestions’ frequencies, scores, and ranks were statistically evaluated.

#### Feature Extraction and Vector Construction

All images were labeled by class (syndromic vs healthy). Vectors were built to hold an attribute for any of the syndromes suggested at least once in DeepGestalt’s top 30 suggestions. To construct a vector for a given photo, the 30 highest Gestalt scores were assigned to their respective attributes; and the remaining attributes were set to 0 (s. matrix.txt in Multimedia Appendix 1).

#### Classification

To differentiate between syndromic and healthy portrait photos, we trained linear support vector machines (SVMs) using the LinearSVM class of scikit-learn, version 0.21.3, with default parameters in Python 3.7. To avoid overfitting, training and testing were performed using a leave-1-out classification scheme. Since ethnic background is a possible confounder of DeepGestalt [15,22,26,29,33], we designed classification experiments based on all images, images of White persons, and those of persons with other ethnicities, to benchmark the influence of ethnicity on SVM performance.

To test a possible influence of the number of top ranks considered, classification of all images was run 30 times with the number of considered top Gestalt ranks, ranging from 1 to 30.

#### Statistical Analysis

Scores of the syndromic and healthy control cohort were tested to be different using a 2-sided, independent Welch *t* test. Difference of receiver operating characteristics (ROCs) was tested using a DeLong test. Classification performance was assessed using Matthews correlation coefficient (MCC). All statistical tests were performed in Python 3.7; the code can be found in Multimedia Appendix 1.

#### Data and Code Availability

**Figure 1:**
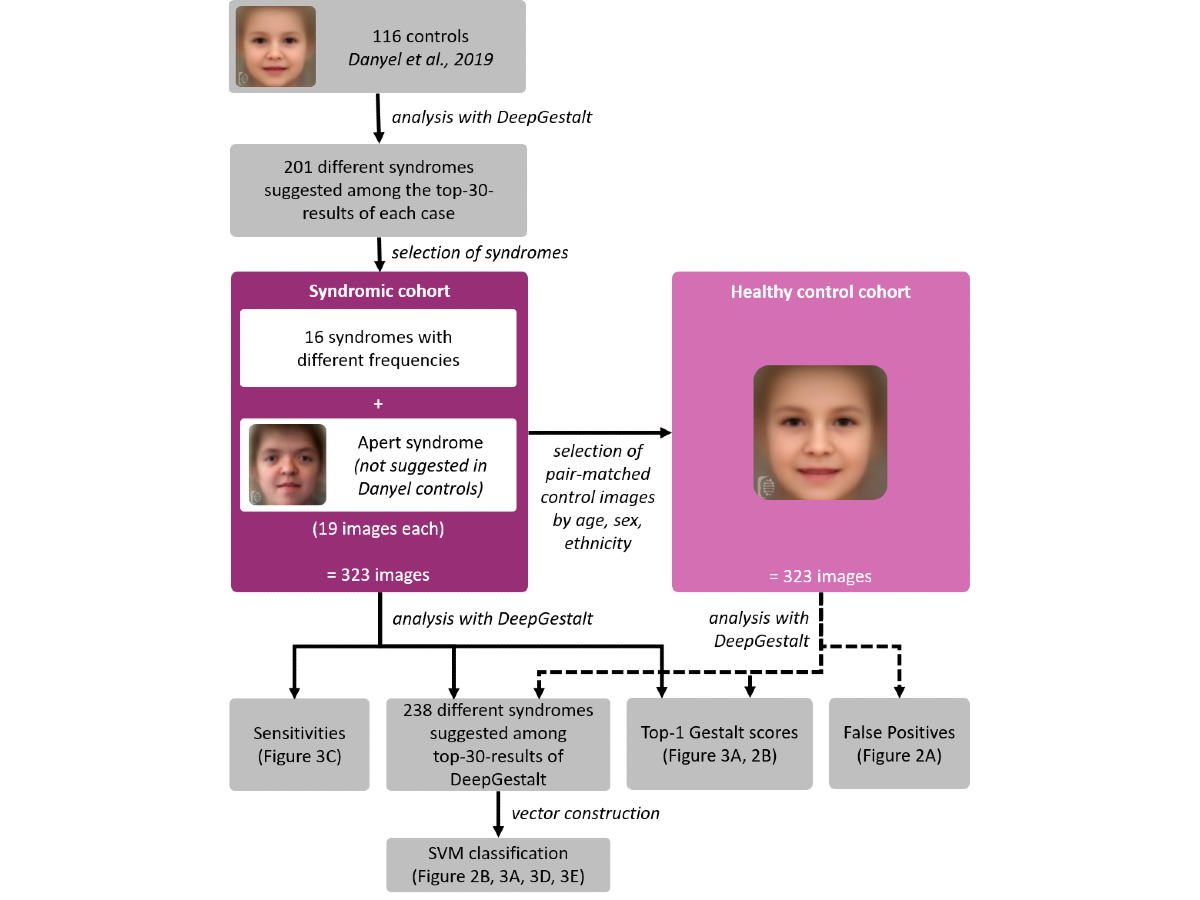
Workflow of classification experiments.

The data and code can be found in Multimedia Appendix 1. For reasons of data protection, all data were cumulated (where possible), deidentified, and minimized. Facial images depicted in Figure 1 show computer-generated composite masks and not real individuals. In Multimedia Appendix 1, file data.txt describes the diagnosis, age, sex, and ethnicity of persons in the analyzed set of images; and file matrix.txt contains DeepGestalt’s output vectors as used for this study. Files differentiator.py and reproduce.py may be used for reproducing the statistical results of this study. Further information may be found in file readme.txt (Multimedia Appendix 1).

## Results

### Included Images

We could include 19 images for each of the 17 syndromes in the syndromic cohort. A total of 83% (272/323) of these images were of White persons (file data.txt of Multimedia Appendix 1). Images from the syndromic cohort were matched to 323 images forming the matched control cohort, resulting in a total number of 646 analyzed photos (Figure 1).

### Frequencies and Scores of Suggested Syndromes in Control Individuals

**Figure 2:**
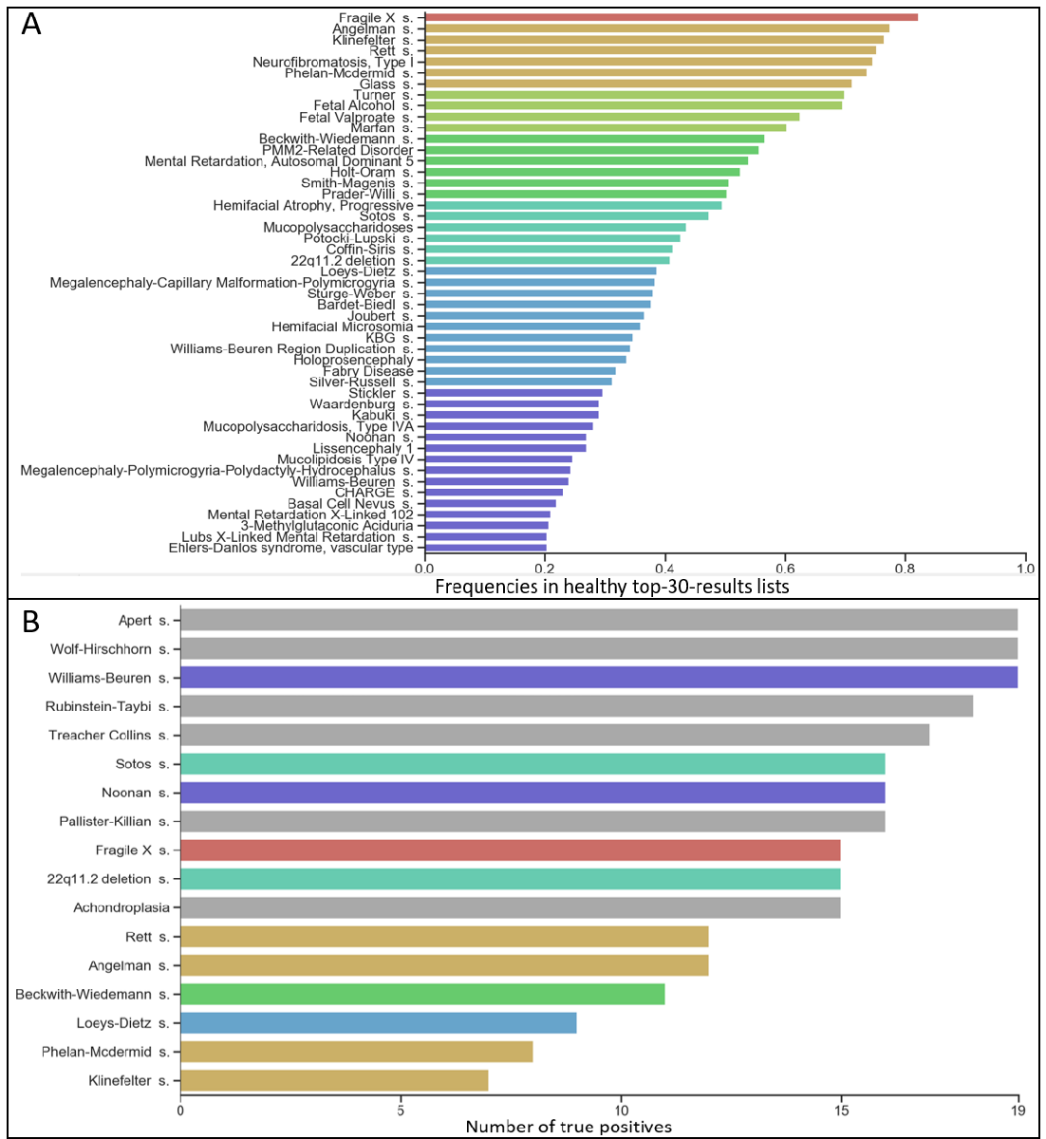
(A) Frequency of syndromes suggested by DeepGestalt in more than 20% of the matched control cohort’s top-30-results lists. Colors indicate frequency percentages. (B) Number of images correctly classified as “syndromic”; colors relate to (A) and gray indicates <20%.

DeepGestalt suggested 238 different syndromes among the top 30 suggestions of the matched control cohort. One syndrome was suggested in more than 80% of the cases (Fragile X syndrome, 82%), 6 syndromes in 70%-80% of the cases; 4 syndromes in 60%-70% of the cases; 6 syndromes in 50%-60% of the cases; 6 syndromes in 40%-50% of the cases; 11 syndromes in 30%-40% of the cases; 15 syndromes in 20%-30% of the cases; 29 syndromes in 10%-20% of the cases; and 160 syndromes at least once in less than 10% of the cases (Figure 2A).

**Figure 3:**
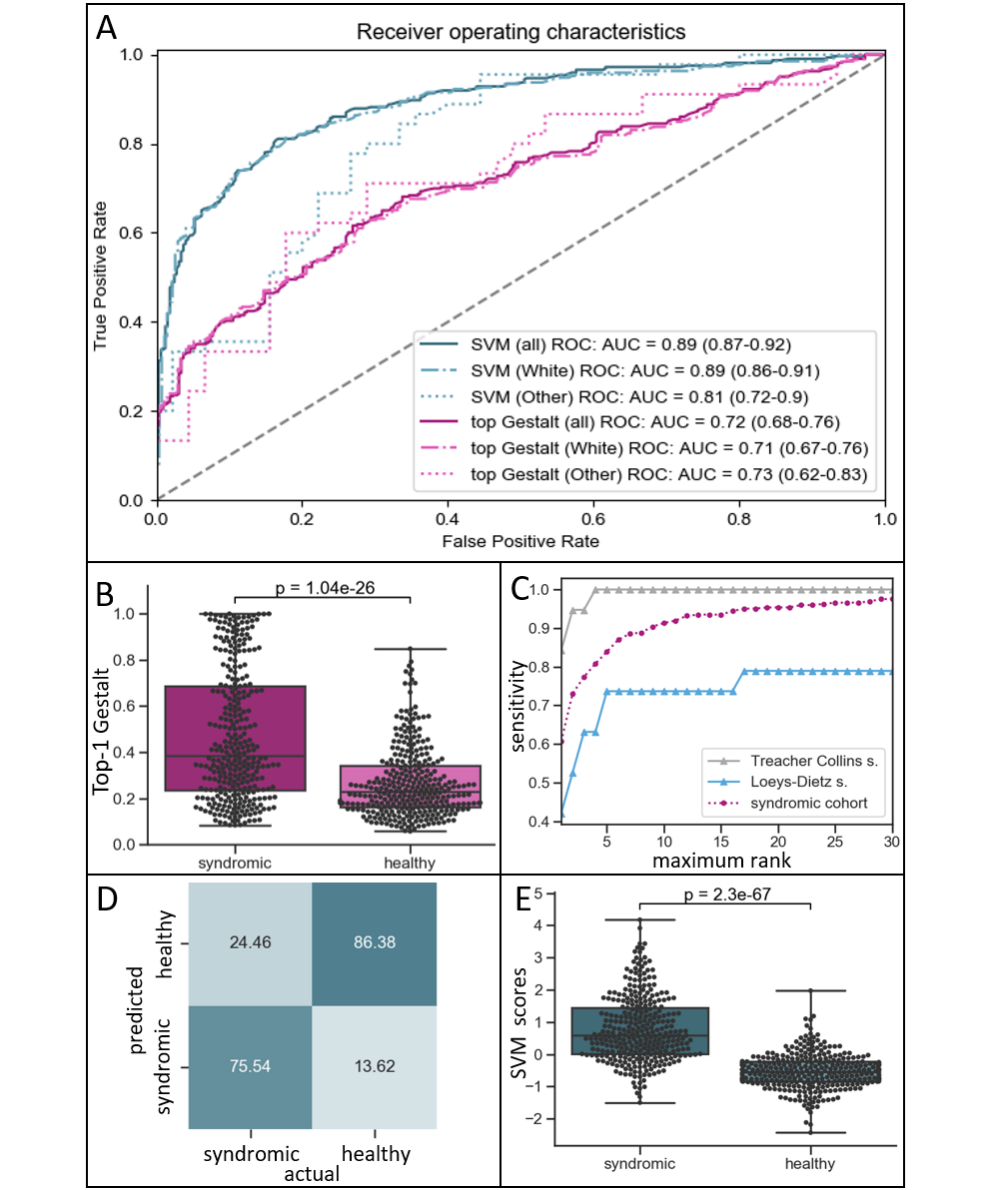
(A) Receiver operating characteristic (ROC) curves: dashed line indicates random ROC curve; note that support vector machine (SVM) scores yield higher areas under the ROC curves (AUROCs) than their respective raw first-rank Gestalt scores. (B) Distribution of first-rank Gestalt scores in the syndromic cohort and the matched control cohort (healthy). (C) Sensitivities of DeepGestalt (X-axis: number of considered top ranks). Dark-purple circles: average of syndromic cohort; gray triangles: 19 images with Treacher-Collins syndrome; blue triangles: 19 images with Loeys-Dietz syndrome. (D) Distribution of SVM scores in the syndromic cohort and the matched control cohort; note: improved separability as compared to B. (E) SVM classification results based on the entire matched control cohort and syndromic cohort (threshold SVM score: 0).

The highest first-rank Gestalt score of the matched control cohort amounted to 0.85, and the lowest, to 0.06, with a mean of 0.27 (SD 0.15). First-rank Gestalt scores of the syndromic cohort (highest 1.0; lowest 0.08; mean 0.47, SD 0.28) and the matched control cohort appeared to be separable with an AUROC of 0.72 (95% CI 0.68-0.76) (Figure 3A). Notably, this was found for both tested ethnic groups (Figure 3A, Multimedia Appendix 2), White persons only (AUROC 0.71, 95% CI 0.67-0.76; *P*<.001), and persons of other ethnicities only (AUROC 0.71, 95% CI 0.62-0.83; *P*<.001). Separability of the 2 cohorts is evident and significant (*P*<.001), as shown in Figure 3B.

### Sensitivity of DeepGestalt

DeepGestalt’s average top 10 sensitivity in the syndromic cohort amounted to 91%, varying between the 17 tested syndromes (Figure 3C, Multimedia Appendix 3). Interestingly, DeepGestalt was sensitive independent of ethnicity (White persons only, 90%; persons of other ethnicities only, 97%). A total of 7 syndromes reached a top 10 sensitivity of 100% (Fragile X, Noonan, Phelan-McDermid, Rett, Sotos, Treacher-Collins, and Williams-Beuren syndromes). DeepGestalt performed worst for Loeys-Dietz syndrome, with a top 10 sensitivity of 74% (Figure 3C).

### Performance of the SVM

Sensitivities of binary SVM classification differed between syndromes (Figure 2B). All images of individuals with Apert syndrome, Wolf-Hirschhorn syndrome, and Williams-Beuren syndrome were correctly classified as being syndromic. The SVM performed worst on the 19 images of individuals with Klinefelter syndrome, correctly classifying only 7 of them as syndromic.

Binary SVM classification of DeepGestalt’s output achieved an increased separability of syndromic images and healthy controls as compared to top Gestalt scores with an AUROC of 0.89 (95% CI 0.87-0.92) (Figure 3A). Again, this was true in both tested ethnic groups (Figure 3A), for photos of White persons (AUROC 0.88, 95% CI 0.86-0.91; *P*<.001) and those of persons of other ethnicities (AUROC 0.79, 95% CI 0.62-0.83). However, difference in ROCs was not significant in the latter (*P*=.13). SVM classification performance improved with an increasing number of considered ranks. Using the top 30 Gestalt scores showed the best MCC (0.63), as shown in Multimedia Appendix 4, with a sensitivity of 75.54% and a specificity of 86.38% (Figure 3D). Separability was significant (*P*<.001) (Figure 3E).

## Discussion

### Classification of Images of Individuals Without a Genetic Syndrome

To our knowledge, this is the first study to systematically analyze DeepGestalt’s behavior on portrait photos of individuals without a genetic syndrome. For these images, we show that DeepGestalt’s syndrome suggestions follow an interesting distribution. Certain syndromes are implied as differential diagnoses with a considerably high likelihood. Among these were Fragile X, Klinefelter, Rett, and Angelman syndromes, which were suggested in more than 3 quarters of the matched control cohort. In contrast, syndromes such as Treacher-Collins syndrome and Wolf-Hirschhorn syndrome were implied very rarely.

DeepGestalt cannot assign the class label “inconspicuous.” Yet, DeepGestalt’s scores are used to help judge the presence of a given syndrome. Based on a high maximum Gestalt score, a user could assume that the individual depicted in an entered image is likely to have a syndrome. Likewise, one is tempted to assume that a low maximum Gestalt score makes an underlying syndrome unlikely. Indeed, the mean of first-rank Gestalt scores is higher in images depicting syndromic facies than in images of individuals without a genetic syndrome. Similarly, scores higher than 0.85 appear to be specific indicators of a syndromic facies, and those lower than 0.08 are not suggestive of a genetic syndrome. However, these specific values are very rare. Gestalt scores alone are only fairly sufficient for judging the presence or absence of a genetic syndrome with facial dysmorphism since the distributions of the highest Gestalt scores of the syndromic and matched control cohort greatly overlap. We show that this problem can be reduced by considering both top Gestalt scores and the actual list of suggested syndrome matches. The boost in discriminatory power is illustrated by the increase of the respective AUROCs. Although DeepGestalt cannot directly assess the presence/absence of a syndromic facies, machine learning–based tools (eg, SVMs) built on top of DeepGestalt may be used for this purpose.

It is noteworthy that we achieved promising results with a comparably low number of samples and a low complexity classification model with default hyperparameters. We assume that the quality and complexity of future classifiers will improve as more data will become available. Increasing the number of top ranks considered for vector construction increased the performance of the SVM. However, the number of DeepGestalt’s suggestions accessible via Face2Gene CLINIC is limited to 30 suggestions. We hypothesize that using more than just the 30 top ranks for vector construction might further boost classification performance. We classified DeepGestalt’s output to predict the presence of a syndromic facies. We also suggest evaluating classification performance based on DeepGestalt’s input vectors.

### Potential Confounders

Until now, differences in the diagnostic performance of DeepGestalt, which arise due to the ethnicity of the person depicted, have been evaluated using DeepGestalt's sensitivity. Studies of earlier versions of DeepGestalt showed that its sensitivity is dependent on the ethnic background in certain syndromes [15,22]. Studies of more recent versions of DeepGestalt suggested that ethnicity had no major influence on its sensitivity [26,29]. In our set of syndromic images, DeepGestalt’s sensitivity is remarkably high, which is in line with the previous studies highlighting DeepGestalt’s good general sensitivity [11,36,42]. This high sensitivity of DeepGestalt was confirmed for both groups of images, those of White persons and those of persons of other ethnicities. Improvement of distinguishability of images of individuals with and without a genetic syndrome appeared to be stronger in the group of photos of White persons than in the group of photos of persons of other ethnicities. However, we assume that this is caused by the limited sample size of images of non-White persons in our data set. We believe that our approach is also applicable to populations comprising predominantly other ethnicities.

The SVM had difficulties classifying images of patients with syndromes that were frequently suggested in healthy controls. Possible explanations for DeepGestalt’s output to be similar in controls and individuals with these syndromes could be as follows: (1) such syndromes have only mild characteristic facial features; (2) they have a typical facial gestalt, which is present only in some but not all affected individuals; or (3) they have no typical facies at all. For example, not all patients with Loeys-Dietz syndrome exhibit distinctive facial features [43], and the facial appearances of males with Klinefelter syndrome show no commonly observed characteristics [44].

### Further Research

Further research is necessary to determine DeepGestalt’s capacity to distinguish individuals with and without a genetic syndrome when combined with other sources of information, such as genetic test results and nonfacial phenotypic information. We suggest including additional scores that are based on both phenotype and genotype (eg, prioritization of exome data by image analysis [PEDIA] scores [42]) in future classifiers of the presence/absence of a syndromic facies.

The increasing use and quality of facial phenotyping software in clinical genetics should also be accompanied by an ethical evaluation of these systems [45]. This affects issues such as the automation of medical diagnostic action, the sharing of (potentially identifiable) data, and a potentially altered doctor-patient relationship. In particular, a systematic analysis of the patient perspective on the use of computer-aided facial analysis methodologies in clinical genetics is lacking so far.

We believe that our findings will help improve future versions of DeepGestalt and similar systems and are crucial when interpreting Face2Gene’s results in the clinical routine. In particular, we recommend providing users with the false-positive rates of each suggested syndrome.

### Conclusion

DeepGestalt is a computer-aided facial phenotyping tool that showed promising results for detecting a potentially syndromic facies. It yields higher first-rank scores in individuals with a genetic syndrome than in those without a diagnosis of a genetic syndrome. Its output may be classified to improve this detection. The exact stage to use DeepGestalt during the diagnostic makeup of individuals with a suspected genetic syndrome remains to be determined. Primarily, it should be used by expert geneticists.

## Appendix

Multimedia Appendix 1 Code and data.

Multimedia Appendix 2 (A) Distribution of first-rank Gestalt scores for the images of White persons in the syndromic cohort and the matched control cohort (healthy). (B) Distribution of first-rank Gestalt scores for the images of persons with other ethnicities in the syndromic cohort and the matched control cohort (healthy).

Multimedia Appendix 3 DeepGestalt’s sensitivities: purple circles indicate the average of the entire syndromic cohort; for other symbols/coloring, see respective subfigure title.

Multimedia Appendix 4 Performance of the SVM on the entire syndromic cohort and matched control cohort: X-axis number of top-rank Gestalt score used for vector construction per case. MCC: Matthews correlation coefficient. Note: rising tendency.

## Abbreviations

AUROC: area under the receiver operating characteristic
MCC: Matthews correlation coefficient
PEDIA: prioritization of exome data by image analysis
ROC: receiver operating characteristic
SVM: support vector machine
