# Supplementary figures and images for: Efficiency of Computer-Aided Facial Phenotyping (DeepGestalt) in Individuals With and Without a Genetic Syndrome: Diagnostic Accuracy Study
Source: J Med Internet Res. 2020 Oct 22;22(10):e19263. doi: 10.2196/19263 (PMC7644377; doi:10.2196/19263)

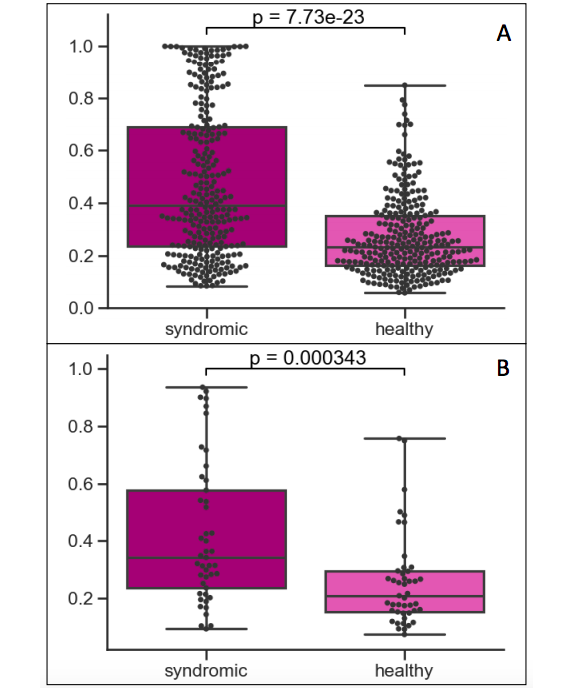

Supplement: Multimedia Appendix 2 [file jmir_v22i10e19263_app2.png]

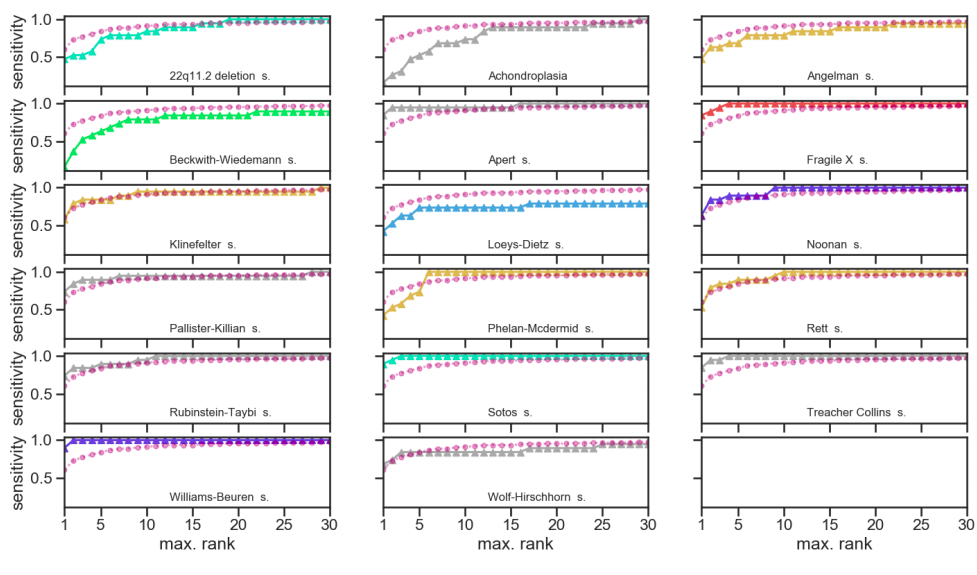

Supplement: Multimedia Appendix 3 [file jmir_v22i10e19263_app3.png]

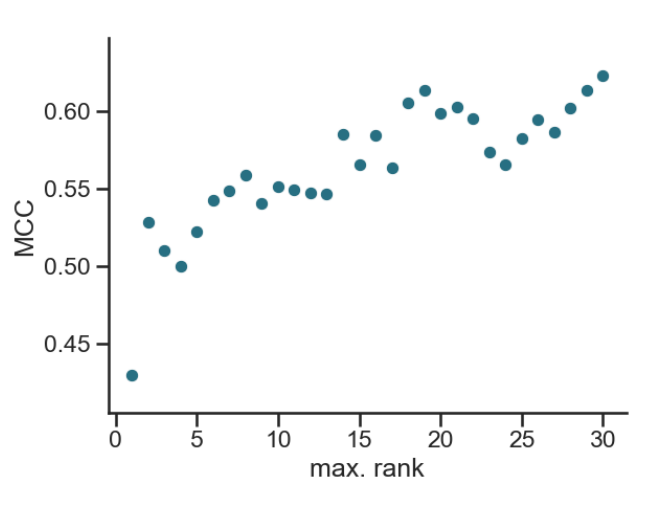

Supplement: Multimedia Appendix 4 [file jmir_v22i10e19263_app4.png]
